# Supplementary material for: Conditional Variable Screening for Ultra‐High Dimensional Longitudinal Data With Time Interactions
Source: Biom J. 2024 Nov 23;66(8):e70005. doi: 10.1002/bimj.70005 (PMC11585226; doi:10.1002/bimj.70005)
Supplement: Supplementary file 1 — Supporting Information [file BIMJ-66-e70005-s002.pdf]

Supporting information for ”Conditional variable screening for ultra-high dimensional longitudinal data with time interactions” by Andrea Bratsberg, Abhik Ghosh and Magne Thoresen

A Example 1

This appendix contains some further simulation studies. We consider the same example as Example 1 in the article, but we consider the cases where either the two sets are disjoint or they are identical.

A.1 $\mathcal{M}_s = \{1, 2, 3, 4\}$ and $\mathcal{I}_s = \{5, 6, 7\}$

Table 1: Table of $r_{\mathcal{M}}$, $r_{\mathcal{I}}$, $\bar{R}_{\mathcal{M}}$ and $\bar{R}_{\mathcal{I}}$ for 400 simulations, together with the 50%, 75%, and 95% percentiles of the minimum model size and the average runtime in seconds in Example 1 (with Red Hat Enterprise Linux 7 machine).

n	Method	$r_{\mathcal{M}}$	$\bar{R}_{\mathcal{M}}$	$r_{\mathcal{I}}$	$\bar{R}_{\mathcal{I}}$	Time	50%	75%	95%
				$(\sigma_b, \sigma_\epsilon)$ = (0.1,0.1)					
40	SIS	0.338	0.776	0.115	0.492	0.034	317	570	918
	GEES.ar1	0.338	0.777	0.112	0.492	0.073	320	568	915
	BCor-SIS	0.045	0.507	0.005	0.231	0.725	644	812	956
	CDC-SIS	0.225	0.711	0.352	0.711	0.823	215	345	678
	LS.intercept	0.005	0.288	0.932	0.978	2.485	274	408	642
	LS.slope	0.455	0.838	0.868	0.955	3.326	69	154	476
80	SIS	0.945	0.986	0.675	0.877	0.081	62	153	559
	GEES.ar1	0.943	0.986	0.672	0.877	1.082	62	156	557
	BCor-SIS	0.605	0.887	0.138	0.536	2.998	392	628	897
	CDC-SIS	0.948	0.987	0.948	0.983	5.165	24	47	173
	LS.intercept	0.608	0.883	1	1	2.743	85	132	281
	LS.slope	0.978	0.994	1	1	3.802	8	11	43
100	SIS	0.980	0.995	0.828	0.941	0.093	29	89	312
	GEES.ar1	0.980	0.995	0.828	0.941	1.361	29	88	307
	BCor-SIS	0.833	0.956	0.268	0.658	5.104	260	467	843
	CDC-SIS	0.99	0.998	0.993	0.998	10.72	12	22	62
	LS.intercept	0.892	0.972	1	1	2.941	47	82	156
	LS.slope	1	1	1	1	4.132	7	7	14
				$(\sigma_b, \sigma_\epsilon)$ = (0.9,0.1)					
40	SIS	0.040	0.469	0.015	0.278	0.039	641	850	963
	GEES.ar1	0.040	0.472	0.015	0.277	0.077	638	849	965
	BCor-SIS	0	0.246	0.005	0.129	0.768	808	918	981
	CDC-SIS	0.022	0.413	0.085	0.422	0.818	490	695	916
	LS.intercept	0	0.127	0.935	0.978	2.872	492	677	893
	LS.slope	0.025	0.425	0.855	0.951	3.652	330	623	859
80	SIS	0.535	0.854	0.278	0.630	0.071	293	571	925
	GEES.ar1	0.540	0.856	0.270	0.629	1.058	291	570	920
	BCor-SIS	0.117	0.574	0.025	0.312	3.066	693	820	962
	CDC-SIS	0.482	0.832	0.735	0.903	5.229	142	282	704
	LS.intercept	0.068	0.514	1	1	2.929	260	397	662
	LS.slope	0.517	0.849	1	1	3.991	86	199	621
100	SIS	0.772	0.938	0.432	0.762	0.108	186	406	808
	GEES.ar1	0.772	0.938	0.435	0.761	1.329	187	404	806
	BCor-SIS	0.228	0.691	0.078	0.412	4.906	578	764	954
	CDC-SIS	0.650	0.902	0.910	0.970	10.79	85	191	568
	LS.intercept	0.228	0.698	1	1	3.048	192	300	511
	LS.slope	0.720	0.926	1	1	4.261	42	130	373

A.2 $\mathcal{M}_s = \mathcal{I}_s = \{1, 2, 3, 4\}$

Table 2: Table of $r_{\mathcal{M}}$, $r_{\mathcal{I}}$, $\bar{R}_{\mathcal{M}}$ and $\bar{R}_{\mathcal{I}}$ for 400 simulations, together with the 50%, 75%, and 95% percentiles of the minimum model size and the average runtime in seconds in Example 1 (with Red Hat Enterprise Linux 7 machine).

n	Method	$r_{\mathcal{M}}$	$\bar{R}_{\mathcal{M}}$	$r_{\mathcal{I}}$	$\bar{R}_{\mathcal{I}}$	Time	50%	75%	95%
				$(\sigma_b, \sigma_\epsilon)$ = (0.1,0.1)					
40	SIS	0.655	0.907	0.655	0.907	0.032	26	71	251
	GEES.ar1	0.652	0.907	0.652	0.907	0.077	26	71	248
	BCor-SIS	0.110	0.620	0.110	0.620	0.748	249	438	796
	CDC-SIS	0.532	0.868	0.532	0.868	0.853	40	124	504
	LS.intercept	0.642	0.906	0.642	0.906	2.505	24	75	256
	LS.slope	0.455	0.836	0.455	0.836	3.222	52	130	392
80	SIS	0.998	0.999	0.998	0.999	0.072	4	5	17
	GEES.ar1	0.998	0.999	0.998	0.999	1.046	4	5	17
	BCor-SIS	0.820	0.953	0.820	0.953	2.995	22	63	270
	CDC-SIS	0.985	0.996	0.985	0.996	5.201	4	6	22
	LS.intercept	0.998	0.999	0.998	0.999	2.726	4	5	15
	LS.slope	0.985	0.996	0.985	0.996	3.630	4	7	38
100	SIS	1	1	1	1	0.106	4	4	6
	GEES.ar1	1	1	1	1	1.420	4	4	6
	BCor-SIS	0.955	0.988	0.955	0.988	4.983	7	20	96
	CDC-SIS	1	1	1	1	9.975	4	4	7
	LS.intercept	1	1	1	1	2.858	4	4	6
	LS.slope	1	1	1	1	3.888	4	4	10
				$(\sigma_b, \sigma_\epsilon)$ = (0.9,0.1)					
40	SIS	0.328	0.766	0.328	0.766	0.038	92	204	561
	GEES.ar1	0.330	0.767	0.330	0.767	0.080	94	205	565
	BCor-SIS	0.015	0.431	0.015	0.431	0.733	397	638	904
	CDC-SIS	0.250	0.740	0.250	0.740	0.837	102	266	721
	LS.intercept	0.627	0.899	0.627	0.899	2.750	27	76	259
	LS.slope0.442	0.832	0.442	0.832	3.510	57	138	391	
80	SIS	0.945	0.986	0.945	0.986	0.081	7	20	93
	GEES.ar1	0.945	0.986	0.945	0.986	1.040	7	19	94
	BCor-SIS	0.460	0.824	0.460	0.824	3.022	100	268	648
	CDC-SIS	0.938	0.984	0.938	0.984	5.796	8	19	106
	LS.intercept	0.998	0.999	0.998	0.999	2.860	4	6	18
	LS.slope	0.985	0.996	0.985	0.996	3.938	5	8	33
100	SIS	0.990	0.998	0.990	0.998	0.084	5	8	27
	GEES.ar1	0.990	0.998	0.990	0.998	1.326	5	8	27
	BCor-SIS	0.698	0.917	0.698	0.917	4.927	50	133	421
	CDC-SIS	0.980	0.995	0.980	0.995	9.838	5	8	36
	LS.intercept	1	1	1	1	2.946	4	4	7
	LS.slope	1	1	1	1	4.161	4	4	11

B Example 2

This section contains some further simulations for Example 2 in the main text, with either disjoint or identical active sets of main effects and variables.

B.1 $\mathcal{M}_s = \{1, 2, 3, 4\}$ and $\mathcal{I}_s = \{5, 6, 7\}$

Table 3: Table of $r_{\mathcal{M}}$, $r_{\mathcal{I}}$, $\bar{R}_{\mathcal{M}}$ and $\bar{R}_{\mathcal{I}}$ for 400 simulations, together with the 50%, 75%, and 95% percentiles of the minimum model size and the average runtime in seconds in Example 2 (with Red Hat Enterprise Linux 7 machine).

n	Method	$r_{\mathcal{M}}$	$\bar{R}_{\mathcal{M}}$	$r_{\mathcal{I}}$	$\bar{R}_{\mathcal{I}}$	Time	50%	75%	95%
				$(\sigma_b, \sigma_\epsilon)$ = (0.1,0.1)					
40	SIS	0.718	0.927	0.325	0.709	0.033	150	312	747
	GEES.cs	0.752	0.931	0.460	0.773	0.072	105	256	632
	GEES.ar1	0.925	0.981	0.657	0.867	0.067	47	121	423
	BCor-SIS	0.320	0.774	0.045	0.406	0.852	456	718	925
	CDC-SIS	0.268	0.751	0.305	0.676	0.818	228	408	735
	LS.intercept	0.672	0.907	0.880	0.959	2.503	49	97	300
	LS.slope	0.990	0.998	0.950	0.983	2.940	10	22	70
80	SIS	1	1	0.897	0.964	0.078	18	46	219
	GEES.cs	0.998	0.999	0.958	0.986	1.066	12	25	114
	GEES.ar1	1	1	0.993	0.998	1.075	8	12	42
	BCor-SIS	0.955	0.988	0.442	0.774	3.469	154	331	719
	CDC-SIS	0.965	0.991	0.905	0.968	5.149	29	69	239
	LS.intercept	1	1	1	1	2.783	7	10	20
	LS.slope	1	1	1	1	3.232	7	7	7
100	SIS	1	1	0.985	0.995	0.096	10	22	93
	GEES.cs	1	1	0.998	0.999	1.314	8	12	37
	GEES.ar1	1	1	1	1	1.254	7	8	14
	BCor-SIS	0.995	0.999	0.662	0.874	5.677	80	213	563
	CDC-SIS	0.998	0.999	0.983	0.994	9.853	14	27	94
	LS.intercept	1	1	1	1	2.943	7	7	10
	LS.slope	1	1	1	1	3.405	7	7	7
				$(\sigma_b, \sigma_\epsilon)$ = (0.9,0.1)					
40	SIS	0.007	0.366	0.013	0.222	0.031	774	912	982
	GEES.cs	0.025	0.384	0.013	0.232	0.078	766	900	980
	GEES.ar1	0.043	0.462	0.030	0.297	0.061	712	877	976
	BCor-SIS	0	0.266	0.002	0.104	0.862	843	922	987
	CDC-SIS	0	0.191	0.007	0.164	0.816	773	905	982
	LS.intercept	0	0.228	0.043	0.340	2.864	692	858	966
	LS.slope	0.825	0.954	0.652	0.866	3.308	54	114	344
80	SIS	0.240	0.701	0.132	0.498	0.063	554	774	974
	GEES.cs	0.332	0.738	0.190	0.557	0.991	498	762	961
	GEES.ar1	0.440	0.807	0.245	0.618	0.951	426	651	912
	BCor-SIS	0.112	0.589	0.015	0.255	3.482	761	877	968
	CDC-SIS	0.072	0.515	0.082	0.416	5.181	626	761	933
	LS.intercept	0.160	0.632	0.342	0.702	2.974	394	573	884
	LS.slope	1	1	0.995	0.998	3.697	8	9	31
100	SIS	0.470	0.829	0.215	0.593	0.097	471	691	943
	GEES.cs	0.542	0.849	0.278	0.655	1.342	376	679	887
	GEES.ar1	0.682	0.909	0.392	0.724	1.220	310	519	864
	BCor-SIS	0.240	0.703	0.020	0.312	5.774	722	859	955
	CDC-SIS	0.212	0.68	0.125	0.52	9.681	570	741	916
	LS.intercept	0.390	0.788	0.525	0.800	3.061	286	484	772
	LS.slope	1	1	1	1	3.769	7	7	11

B.2 $\mathcal{M}_s = \mathcal{I}_s = \{1, 2, 3, 4\}$

Table 4: Table of $r_{\mathcal{M}}$, $r_{\mathcal{I}}$, $\bar{R}_{\mathcal{M}}$ and $\bar{R}_{\mathcal{I}}$ for 400 simulations, together with the 50%, 75%, and 95% percentiles of the minimum model size and the average runtime in seconds in Example 2 (with Red Hat Enterprise Linux 7 machine).

n	Method	$r_{\mathcal{M}}$	$\bar{R}_{\mathcal{M}}$	$r_{\mathcal{I}}$	$\bar{R}_{\mathcal{I}}$	Time	50%	75%	95%
				$(\sigma_b, \sigma_\epsilon) = (0.1,0.1)$					
40	SIS	0.960	0.990	0.960	0.990	0.033	6	11	53
	GEES.cs	0.930	0.981	0.930	0.981	0.072	5	8	76
	GEES.ar1	0.995	0.999	0.995	0.999	0.070	4	4	10
	BCor-SIS	0.660	0.905	0.660	0.905	0.917	32	91	304
	CDC-SIS	0.665	0.911	0.665	0.911	0.827	30	88	232
	LS.intercept	0.985	0.996	0.985	0.996	2.529	4	6	24
	LS.slope	1	1	1	1	2.964	4	4	5
80	SIS	1	1	1	1	0.078	4	4	4
	GEES.cs	1	1	1	1	1.027	4	4	4
	GEES.ar1	1	1	1	1	1.046	4	4	4
	BCor-SIS	0.998	0.999	0.998	0.999	3.589	4	5	17
	CDC-SIS	1	1	1	1	5.169	4	5	15
	LS.intercept	1	1	1	1	2.760	4	4	4
	LS.slope	1	1	1	1	3.333	4	4	4
100	SIS	1	1	1	1	0.112	4	4	4
	GEES.cs	1	1	1	1	1.258	4	4	4
	GEES.ar1	1	1	1	1	1.325	4	4	4
	BCor-SIS	1	1	1	1	5.720	4	4	5
	CDC-SIS	1	1	1	1	10.36	4	4	6
	LS.intercept	1	1	1	1	3.053	4	4	4
	LS.slope	1	1	1	1	3.580	4	4	4
				$(\sigma_b, \sigma_\epsilon) = (0.9,0.1)$					
40	SIS	0.240	0.704	0.240	0.704	0.038	150	304	720
	GEES.cs	0.318	0.735	0.318	0.735	0.080	112	310	752
	GEES.ar1	0.465	0.821	0.465	0.821	0.070	64	152	533
	BCor-SIS	0.043	0.493	0.043	0.493	0.885	386	642	926
	CDC-SIS	0.035	0.451	0.035	0.451	0.819	338	600	861
	LS.intercept	0.225	0.694	0.225	0.694	2.875	136	260	568
	LS.slope	0.995	0.999	0.995	0.999	3.306	4	5	17
80	SIS	0.858	0.963	0.858	0.963	0.075	19	55	310
	GEES.cs	0.897	0.973	0.897	0.973	1.037	9	33	233
	GEES.ar1	0.960	0.990	0.960	0.990	1.016	6	13	93
	BCor-SIS	0.517	0.846	0.517	0.846	3.508	101	261	646
	CDC-SIS	0.545	0.86	0.545	0.86	5.18	100	231	515
	LS.intercept	0.897	0.973	0.897	0.973	2.925	20	53	196
	LS.slope	1	1	1	1	3.424	4	4	4
100	SIS	0.973	0.993	0.973	0.993	0.115	10	25	85
	GEES.cs	0.960	0.990	0.960	0.990	1.458	5	11	100
	GEES.ar1	0.995	0.999	0.995	0.999	1.269	4	6	22
	BCor-SIS	0.750	0.932	0.750	0.932	5.689	50	137	407
	CDC-SIS	0.76	0.937	0.76	0.937	9.818	60	139	361
	LS.intercept	0.970	0.993	0.970	0.993	3.062	9	19	75
	LS.slope	1	1	1	1	3.755	4	4	4

C Example 3

This appendix contains further simulations for Example 3 in the main text, with disjoint or identical active sets of main and interaction variables.

C.1 $\mathcal{M}_s = \{1, 2, 3, 4\}$ and $\mathcal{I}_s = \{5, 6, 7\}$

Table 5: Table of $r_{\mathcal{M}}$, $r_{\mathcal{I}}$, $\bar{R}_{\mathcal{M}}$ and $\bar{R}_{\mathcal{I}}$ for 400 simulations, together with the 50%, 75%, and 95% percentiles of the minimum model size and the average runtime in seconds in Example 3 (with Red Hat Enterprise Linux 7 machine).

n	Method	$r_{\mathcal{M}}$	$\bar{R}_{\mathcal{M}}$	$r_{\mathcal{I}}$	$\bar{R}_{\mathcal{I}}$	Time	50%	75%	95%
				$(\sigma_b, \sigma_\epsilon)$ = (0.1,0.1)					
40	SIS	0.348	0.783	0.125	0.509	0.030	317	570	918
	GEES.ar1	0.428	0.816	0.062	0.400	0.070	391	674	909
	BCor-SIS	0.038	0.471	0.020	0.313	0.741	628	800	942
	CDC-SIS	0.245	0.725	0.268	0.659	0.662	242	402	727
	LS.intercept	0.007	0.304	0.943	0.981	2.506	274	404	650
	LS.slope	0.062	0.568	0.938	0.979	3.066	170	307	577
80	SIS	0.945	0.986	0.693	0.886	0.079	62	153	559
	GEES.ar1	0.970	0.993	0.500	0.797	1.049	116	276	779
	BCor-SIS	0.517	0.854	0.305	0.694	3.066	310	535	877
	CDC-SIS	0.943	0.986	0.9	0.966	5.361	33	67	242
	LS.intercept	0.642	0.896	1.000	1.000	2.834	83	129	285
	LS.slope	0.917	0.979	1.000	1.000	3.448	32	53	157
100	SIS	0.980	0.995	0.840	0.945	0.093	29	89	312
	GEES.ar1	0.998	0.999	0.688	0.882	1.213	64	170	570
	BCor-SIS	0.767	0.936	0.515	0.802	4.926	174	368	785
	CDC-SIS	0.990	0.998	0.975	0.992	9.306	15	32	98
	LS.intercept	0.922	0.980	1.000	1.000	2.961	45	80	159
	LS.slope	0.983	0.996	1.000	1.000	3.693	16	25	70
				$(\sigma_b, \sigma_\epsilon)$ = (0.9,0.1)					
40	SIS	0.043	0.479	0.015	0.281	0.035	641	850	963
	GEES.ar1	0.048	0.500	0.013	0.221	0.064	685	860	976
	BCor-SIS	0	0.252	0.005	0.158	0.766	790	918	980
	CDC-SIS	0.030	0.418	0.040	0.356	0.658	535	738	915
	LS.intercept	0	0.139	0.935	0.978	2.805	491	666	873
	LS.slope	0	0.186	0.930	0.977	3.374	441	676	876
80	SIS	0.545	0.857	0.285	0.637	0.075	293	571	925
	GEES.ar1	0.575	0.872	0.152	0.504	0.969	400	693	947
	BCor-SIS	0.122	0.577	0.048	0.377	3.087	664	812	948
	CDC-SIS	0.495	0.839	0.547	0.826	4.815	188	317	722
	LS.intercept	0.078	0.531	1.000	1.000	2.933	256	397	651
	LS.slope	0.145	0.646	1.000	1.000	3.604	210	349	644
100	SIS	0.782	0.942	0.442	0.768	0.105	186	406	808
	GEES.ar1	0.812	0.952	0.278	0.632	1.285	300	529	912
	BCor-SIS	0.208	0.682	0.128	0.490	4.910	528	722	945
	CDC-SIS	0.652	0.903	0.810	0.932	9.239	111	225	592
	LS.intercept	0.238	0.711	1	1	2.998	189	299	511
	LS.slope	0.375	0.796	1	1	3.806	146	259	490

C.2 $\mathcal{M}_s = \mathcal{I}_s = \{1, 2, 3, 4\}$

Table 6: Table of $r_{\mathcal{M}}$, $r_{\mathcal{I}}$, $\bar{R}_{\mathcal{M}}$ and $\bar{R}_{\mathcal{I}}$ for 400 simulations, together with the 50%, 75%, and 95% percentiles of the minimum model size and the average runtime in seconds in Example 3 (with Red Hat Enterprise Linux 7 machine).

n	Method	$r_{\mathcal{M}}$	$\bar{R}_{\mathcal{M}}$	$r_{\mathcal{I}}$	$\bar{R}_{\mathcal{I}}$	Time	50%	75%	95%
				$(\sigma_b, \sigma_\epsilon)$ = (0.1,0.1)					
40	SIS	0.662	0.909	0.662	0.909	0.033	26	71	251
	GEES.ar1	0.660	0.909	0.660	0.909	0.062	27	71	246
	BCor-SIS	0.120	0.643	0.120	0.643	0.720	226	426	786
	CDC-SIS	0.547	0.873	0.547	0.873	0.661	40	123	510
	LS.intercept	0.652	0.906	0.652	0.906	2.514	25	72	269
	LS.slope	0.637	0.903	0.637	0.903	3.065	28	76	279
80	SIS	0.998	0.999	0.998	0.999	0.069	4	5	17
	GEES.ar1	0.998	0.999	0.998	0.999	1.032	4	5	17
	BCor-SIS	0.848	0.961	0.848	0.961	3.080	17	52	260
	CDC-SIS	0.985	0.996	0.985	0.996	4.75	4	6	22
	LS.intercept	0.995	0.999	0.995	0.999	2.825	4	5	18
	LS.slope	0.995	0.999	0.995	0.999	3.487	4	5	19
100	SIS	1	1	1	1	0.124	4	4	6
	GEES.ar1	1	1	1	1	1.210	4	4	6
	BCor-SIS	0.968	0.991	0.968	0.991	4.921	6	16	93
	CDC-SIS	1	1	1	1	9.304	4	4	7
	LS.intercept	1	1	1	1	3.021	4	4	6
	LS.slope	1	1	1	1	3.756	4	4	7
				$(\sigma_b, \sigma_\epsilon)$ = (0.9,0.1)					
40	SIS	0.335	0.771	0.335	0.771	0.037	92	204	561
	GEES.ar1	0.282	0.744	0.282	0.744	0.071	102	232	608
	BCor-SIS	0.020	0.452	0.020	0.452	0.765	381	621	889
	CDC-SIS	0.235	0.731	0.235	0.731	0.657	118	279	731
	LS.intercept	0.630	0.899	0.630	0.899	2.873	29	76	281
	LS.slope	0.603	0.889	0.603	0.889	3.438	30	85	365
80	SIS	0.945	0.986	0.945	0.986	0.076	7	20	93
	GEES.ar1	0.935	0.984	0.935	0.984	1.143	9	25	114
	BCor-SIS	0.498	0.849	0.498	0.849	2.993	90	225	651
	CDC-SIS	0.927	0.981	0.927	0.981	4.808	9	21	120
	LS.intercept	0.995	0.999	0.995	0.999	2.891	4	6	19
	LS.slope	0.995	0.999	0.995	0.999	3.669	4	6	18
100	SIS	0.990	0.998	0.990	0.998	0.114	5	8	27
	GEES.ar1	0.990	0.998	0.990	0.998	1.255	5	9	36
	BCor-SIS	0.755	0.933	0.755	0.933	4.896	42	111	377
	CDC-SIS	0.980	0.995	0.980	0.995	9.339	5	9	40
	LS.intercept	1	1	1	1	3.042	4	4	6
	LS.slope	1	1	1	1	3.961	4	4	6

D Example 4

This appendix contains further simulations for Example 4 in the main text, with $n \in \{40, 80, 100\}$ and $\sigma_b \in \{0.1, 0.9\}$.

Table 7: $r_{\mathcal{M}}$ and $r_{\mathcal{I}}$ for different levels of between-variables correlation $\omega \in \{0, 0.5, 0.9\}$ for Example 4 and $\sigma_b = 0.1$.

n	Method	$r_{\mathcal{M}}$			$r_{\mathcal{I}}$		
		$\omega = 0$	$\omega = 0.5$	$\omega = 0.9$	$\omega = 0$	$\omega = 0.5$	$\omega = 0.9$
40	SIS	0.355	0.135	0.080	0.230	0.082	0.062
	GEES.ar1	0.355	0.132	0.080	0.230	0.082	0.062
	BCor-SIS	0.068	0.062	0.035	0.043	0.025	0.032
	CDC-SIS	0.290	0.070	0.043	0.275	0.075	0.065
	LS.intercept	0.080	0.018	0.013	0.642	0.232	0.198
	LS.slope	0.728	0.402	0.280	0.728	0.395	0.285
100	SIS	0.943	0.757	0.743	0.718	0.603	0.532
	GEES.ar1	0.945	0.75	0.745	0.718	0.603	0.535
	BCor-SIS	0.750	0.690	0.635	0.282	0.408	0.358
	CDC-SIS	0.290	0.070	0.043	0.275	0.075	0.065
	LS.intercept	0.573	0.238	0.255	1	0.958	0.938
	LS.slope	1	0.983	0.983	1	0.983	0.983

Table 8: $r_{\mathcal{M}}$ and $r_{\mathcal{I}}$ for different levels of between-variables correlation $\omega \in \{0, 0.5, 0.9\}$ for Example 4 and $\sigma_b = 0.9$.

n	Method	$r_{\mathcal{M}}$			$r_{\mathcal{I}}$		
		$\omega = 0$	$\omega = 0.5$	$\omega = 0.9$	$\omega = 0$	$\omega = 0.5$	$\omega = 0.9$
40	SIS	0.310	0.098	0.022	0.202	0.062	0.035
	GEES.ar1	0.310	0.098	0.022	0.202	0.060	0.035
	BCor-SIS	0.045	0.048	0.007	0.013	0.025	0.015
	CDC-SIS	0.250	0.055	0.013	0.248	0.062	0.025
	LS.intercept	0.070	0.020	0.013	0.642	0.235	0.192
	LS.slope	0.670	0.210	0.025	0.677	0.362	0.208
80	SIS	0.815	0.585	0.402	0.580	0.460	0.275
	GEES.ar1	0.815	0.585	0.405	0.580	0.460	0.272
	BCor-SIS	0.530	0.430	0.212	0.190	0.270	0.128
	CDC-SIS	0.828	0.448	0.298	0.790	0.460	0.268
	LS.intercept	0.358	0.152	0.130	0.998	0.895	0.815
	LS.slope	0.998	0.858	0.218	0.998	0.945	0.875
100	SIS	0.922	0.735	0.605	0.670	0.583	0.428
	GEES.ar1	0.925	0.735	0.605	0.672	0.583	0.428
	BCor-SIS	0.675	0.640	0.462	0.282	0.380	0.248
	CDC-SIS	0.938	0.608	0.505	0.900	0.598	0.455
	LS.intercept	0.550	0.238	0.232	1	0.958	0.930
	LS.slope	1	0.955	0.395	1	0.98	0.978

E Stability selection with SCAD

Table 9: Table of the relative selection frequency of the top selected main variables and interaction variables based on the SCAD method for linear mixed models.

Method	Main variables	Interaction variables
SIS	TNFRSF4 (0.79), BATF3 (0.76), ABCG1 (0.73), LILRA3 (0.73), BLNK (0.69), CD48 (63), ADA (0.61), SMAD3 (0.60)	-
GEES.cs	BLNK (0.87), BATF3 (0.76), ABCG1 (0.83), LILRB4 (0.82), TGFBR2 (0.82), HMGCR (0.81), CTSG (0.73), NFATC1 (0.63), UBE2L3 (0.61), CD80 (0.60)	-
CDC-SIS	TNFRSF4 (0.77), LILRA3 (0.69), CD55 (0.63), ABCG1 (0.63), FCER1A (0.62)	-
