# Supplementary material for: Conditional Variable Screening for Ultra‐High Dimensional Longitudinal Data With Time Interactions
Source: Biom J. 2024 Nov 23;66(8):e70005. doi: 10.1002/bimj.70005 (PMC11585226; doi:10.1002/bimj.70005)
Supplement: Supplementary file 2 — Supporting Information [file BIMJ-66-e70005-s001.zip › code_supplement_bimj.202300154/reproduce_results.html]

Reproduce results


# Reproduce results

### Example 1: table 1

To reproduce Table 1 in the manuscript, simply run the following
command in the terminal (inside the folder “sim\_examples”):

```
Rscript perform_analysis.R ex1 400 filename
```

where the number 400 is the number of reruns. To minimize the running
time this could be lowered. The argument “filename” is the name of the
rds-file that will be saved. Running the script above saves a rds-file
with the results corresponding to table 1. We have attached a file after
running the above command, and reading the file gives the following
table:

```
ex1_results <- readRDS("sim_examples/intermediate_results/ex1_fullrun.rds")
ex1_results[,-seq(1,3)] <- as.numeric(ex1_results[,-seq(1,3)])
kable(ex1_results)
```

| ns | sigmab | Method | recovery A | mean rate A | recovery I | mean rate I | Time | 50% | 75% | 95% |
| --- | --- | --- | --- | --- | --- | --- | --- | --- | --- | --- |
| 40 | 0.1 | GEES.ind | 0.378 | 0.82 | 0.232 | 0.779 | 0.041 | 276 | 587 | 916 |
| 40 | 0.1 | GEES.cs | 0.378 | 0.82 | 0.232 | 0.779 | 0.08 | 276 | 587 | 916 |
| 40 | 0.1 | GEES.ar1 | 0.375 | 0.819 | 0.235 | 0.779 | 0.075 | 270 | 591 | 919 |
| 40 | 0.1 | BCor-SIS | 0.088 | 0.604 | 0.03 | 0.554 | 0.938 | 570 | 804 | 966 |
| 40 | 0.1 | LS.intercept | 0.082 | 0.728 | 0.67 | 0.912 | 2.557 | 248 | 359 | 485 |
| 40 | 0.1 | LS.slope | 0.672 | 0.914 | 0.672 | 0.914 | 3.394 | 18 | 66 | 278 |
| 80 | 0.1 | GEES.ind | 0.863 | 0.966 | 0.58 | 0.895 | 0.071 | 90 | 218 | 728 |
| 80 | 0.1 | GEES.cs | 0.863 | 0.966 | 0.58 | 0.895 | 1.086 | 90 | 218 | 728 |
| 80 | 0.1 | GEES.ar1 | 0.863 | 0.966 | 0.583 | 0.896 | 1.021 | 90 | 216 | 728 |
| 80 | 0.1 | BCor-SIS | 0.583 | 0.891 | 0.23 | 0.796 | 3.028 | 344 | 587 | 880 |
| 80 | 0.1 | LS.intercept | 0.37 | 0.843 | 0.998 | 0.999 | 2.822 | 125 | 187 | 269 |
| 80 | 0.1 | LS.slope | 0.998 | 0.999 | 0.998 | 0.999 | 3.882 | 5 | 5 | 16 |
| 100 | 0.1 | GEES.ind | 0.93 | 0.983 | 0.757 | 0.939 | 0.099 | 48 | 152 | 585 |
| 100 | 0.1 | GEES.cs | 0.93 | 0.983 | 0.757 | 0.939 | 1.282 | 48 | 152 | 585 |
| 100 | 0.1 | GEES.ar1 | 0.932 | 0.983 | 0.76 | 0.94 | 2.337 | 48 | 153 | 588 |
| 100 | 0.1 | BCor-SIS | 0.75 | 0.936 | 0.328 | 0.829 | 4.96 | 282 | 518 | 846 |
| 100 | 0.1 | LS.intercept | 0.613 | 0.903 | 1 | 1 | 2.971 | 94 | 144 | 219 |
| 100 | 0.1 | LS.slope | 1 | 1 | 1 | 1 | 4.057 | 5 | 5 | 6 |
| 40 | 0.9 | GEES.ind | 0.155 | 0.679 | 0.098 | 0.651 | 0.034 | 405 | 688 | 930 |
| 40 | 0.9 | GEES.cs | 0.155 | 0.679 | 0.098 | 0.651 | 0.067 | 405 | 688 | 930 |
| 40 | 0.9 | GEES.ar1 | 0.152 | 0.679 | 0.098 | 0.649 | 0.078 | 408 | 684 | 923 |
| 40 | 0.9 | BCor-SIS | 0.01 | 0.404 | 0.01 | 0.377 | 0.788 | 662 | 839 | 975 |
| 40 | 0.9 | LS.intercept | 0.078 | 0.705 | 0.62 | 0.896 | 2.872 | 289 | 437 | 707 |
| 40 | 0.9 | LS.slope | 0.33 | 0.774 | 0.547 | 0.869 | 3.711 | 88 | 220 | 534 |
| 80 | 0.9 | GEES.ind | 0.705 | 0.925 | 0.42 | 0.853 | 0.081 | 184 | 441 | 850 |
| 80 | 0.9 | GEES.cs | 0.705 | 0.925 | 0.42 | 0.853 | 0.98 | 184 | 441 | 850 |
| 80 | 0.9 | GEES.ar1 | 0.705 | 0.925 | 0.42 | 0.853 | 1.046 | 186 | 444 | 846 |
| 80 | 0.9 | BCor-SIS | 0.28 | 0.754 | 0.11 | 0.699 | 3.043 | 468 | 696 | 948 |
| 80 | 0.9 | LS.intercept | 0.26 | 0.814 | 0.995 | 0.999 | 2.963 | 167 | 264 | 450 |
| 80 | 0.9 | LS.slope | 0.92 | 0.978 | 0.985 | 0.996 | 4.124 | 8 | 18 | 129 |
| 100 | 0.9 | GEES.ind | 0.843 | 0.961 | 0.573 | 0.891 | 0.095 | 120 | 340 | 859 |
| 100 | 0.9 | GEES.cs | 0.843 | 0.961 | 0.573 | 0.891 | 1.615 | 120 | 340 | 859 |
| 100 | 0.9 | GEES.ar1 | 0.843 | 0.961 | 0.575 | 0.892 | 1.748 | 120 | 337 | 858 |
| 100 | 0.9 | BCor-SIS | 0.448 | 0.846 | 0.228 | 0.777 | 4.847 | 428 | 646 | 923 |
| 100 | 0.9 | LS.intercept | 0.432 | 0.858 | 1 | 1 | 3.071 | 136 | 220 | 376 |
| 100 | 0.9 | LS.slope | 0.975 | 0.994 | 1 | 1 | 4.345 | 5 | 9 | 52 |

```
ex1_cdc <- readRDS("sim_examples/intermediate_results/ex1_cdcscreen.rds")
ex1_cdc[,-seq(1,3)] <- as.numeric(ex1_cdc[,-seq(1,3)])
kable(ex1_cdc)
```

| ns | sigmab | Method | recovery A | mean rate A | recovery I | mean rate I | Time | 50% | 75% | 95% |
| --- | --- | --- | --- | --- | --- | --- | --- | --- | --- | --- |
| 40 | 0.1 | CDC-SIS | 0.308 | 0.794 | 0.275 | 0.789 | 0.818 | 217 | 418 | 719 |
| 80 | 0.1 | CDC-SIS | 0.86 | 0.965 | 0.823 | 0.956 | 5.18 | 44 | 116 | 309 |
| 100 | 0.1 | CDC-SIS | 0.96 | 0.99 | 0.91 | 0.978 | 9.959 | 22 | 61 | 196 |
| 40 | 0.9 | CDC-SIS | 0.112 | 0.653 | 0.125 | 0.657 | 0.821 | 382 | 623 | 856 |
| 80 | 0.9 | CDC-SIS | 0.627 | 0.905 | 0.655 | 0.91 | 5.226 | 111 | 241 | 608 |
| 100 | 0.9 | CDC-SIS | 0.775 | 0.944 | 0.83 | 0.958 | 9.959 | 63 | 164 | 462 |

### Example 2: table 2

To reproduce table 2, simply run the command

```
Rscript perform_analysis.R ex2 400 filename
```

instead of ex1. Similarly to above, this gives the following
table:

```
ex2_results <- readRDS("sim_examples/intermediate_results/ex2_fullrun.rds")
ex2_results[,-seq(1,3)] <- as.numeric(ex2_results[,-seq(1,3)])
kable(ex2_results)
```

| ns | sigmab | Method | recovery A | mean rate A | recovery I | mean rate I | Time | 50% | 75% | 95% |
| --- | --- | --- | --- | --- | --- | --- | --- | --- | --- | --- |
| 40 | 0.1 | GEES.ind | 0.665 | 0.914 | 0.45 | 0.861 | 0.039 | 166 | 352 | 794 |
| 40 | 0.1 | GEES.cs | 0.715 | 0.928 | 0.562 | 0.887 | 0.091 | 89 | 257 | 727 |
| 40 | 0.1 | GEES.ar1 | 0.835 | 0.959 | 0.632 | 0.908 | 0.247 | 51 | 169 | 619 |
| 40 | 0.1 | BCor-SIS | 0.448 | 0.841 | 0.168 | 0.76 | 0.961 | 370 | 648 | 900 |
| 40 | 0.1 | LS.intercept | 0.647 | 0.911 | 0.762 | 0.941 | 2.608 | 59 | 175 | 489 |
| 40 | 0.1 | LS.slope | 0.945 | 0.986 | 0.82 | 0.955 | 3.098 | 14 | 51 | 213 |
| 80 | 0.1 | GEES.ind | 0.958 | 0.989 | 0.828 | 0.957 | 0.066 | 29 | 103 | 548 |
| 80 | 0.1 | GEES.cs | 0.988 | 0.997 | 0.897 | 0.974 | 1.024 | 13 | 49 | 227 |
| 80 | 0.1 | GEES.ar1 | 0.993 | 0.998 | 0.932 | 0.983 | 1.09 | 8 | 25 | 176 |
| 80 | 0.1 | BCor-SIS | 0.915 | 0.979 | 0.47 | 0.868 | 3.464 | 149 | 418 | 786 |
| 80 | 0.1 | LS.intercept | 0.98 | 0.995 | 0.985 | 0.996 | 2.816 | 9 | 20 | 96 |
| 80 | 0.1 | LS.slope | 1 | 1 | 0.995 | 0.999 | 3.346 | 5 | 6 | 25 |
| 100 | 0.1 | GEES.ind | 0.993 | 0.998 | 0.917 | 0.979 | 0.1 | 13 | 50 | 273 |
| 100 | 0.1 | GEES.cs | 0.998 | 0.999 | 0.965 | 0.991 | 1.263 | 7 | 17 | 114 |
| 100 | 0.1 | GEES.ar1 | 1 | 1 | 0.975 | 0.994 | 1.262 | 6 | 12 | 82 |
| 100 | 0.1 | BCor-SIS | 0.96 | 0.99 | 0.623 | 0.906 | 5.764 | 99 | 298 | 767 |
| 100 | 0.1 | LS.intercept | 1 | 1 | 0.998 | 0.999 | 3.057 | 6 | 9 | 30 |
| 100 | 0.1 | LS.slope | 1 | 1 | 1 | 1 | 3.602 | 5 | 5 | 7 |
| 40 | 0.9 | GEES.ind | 0.105 | 0.616 | 0.058 | 0.587 | 0.041 | 488 | 734 | 938 |
| 40 | 0.9 | GEES.cs | 0.117 | 0.628 | 0.098 | 0.605 | 0.101 | 503 | 718 | 952 |
| 40 | 0.9 | GEES.ar1 | 0.222 | 0.724 | 0.132 | 0.686 | 0.085 | 400 | 661 | 941 |
| 40 | 0.9 | BCor-SIS | 0.03 | 0.454 | 0.005 | 0.424 | 0.846 | 679 | 826 | 953 |
| 40 | 0.9 | LS.intercept | 0.06 | 0.574 | 0.108 | 0.614 | 2.848 | 440 | 680 | 922 |
| 40 | 0.9 | LS.slope | 0.815 | 0.954 | 0.662 | 0.914 | 3.303 | 50 | 160 | 510 |
| 80 | 0.9 | GEES.ind | 0.57 | 0.884 | 0.382 | 0.834 | 0.076 | 282 | 568 | 915 |
| 80 | 0.9 | GEES.cs | 0.637 | 0.904 | 0.45 | 0.852 | 0.985 | 213 | 510 | 804 |
| 80 | 0.9 | GEES.ar1 | 0.78 | 0.943 | 0.54 | 0.882 | 1.033 | 157 | 370 | 816 |
| 80 | 0.9 | BCor-SIS | 0.338 | 0.776 | 0.16 | 0.701 | 3.497 | 446 | 663 | 922 |
| 80 | 0.9 | LS.intercept | 0.535 | 0.876 | 0.578 | 0.887 | 2.968 | 193 | 394 | 813 |
| 80 | 0.9 | LS.slope | 0.995 | 0.999 | 0.95 | 0.988 | 3.514 | 6 | 13 | 131 |
| 100 | 0.9 | GEES.ind | 0.79 | 0.946 | 0.51 | 0.873 | 0.102 | 208 | 467 | 898 |
| 100 | 0.9 | GEES.cs | 0.838 | 0.958 | 0.593 | 0.894 | 1.359 | 134 | 362 | 856 |
| 100 | 0.9 | GEES.ar1 | 0.89 | 0.973 | 0.65 | 0.911 | 1.266 | 93 | 262 | 780 |
| 100 | 0.9 | BCor-SIS | 0.552 | 0.873 | 0.28 | 0.783 | 5.706 | 437 | 690 | 939 |
| 100 | 0.9 | LS.intercept | 0.785 | 0.946 | 0.757 | 0.938 | 3.127 | 114 | 288 | 670 |
| 100 | 0.9 | LS.slope | 1 | 1 | 0.985 | 0.996 | 3.683 | 5 | 7 | 34 |

```
ex2_cdc <- readRDS("sim_examples/intermediate_results/ex2_cdcscreen.rds")
ex2_cdc[,-seq(1,3)] <- as.numeric(ex2_cdc[,-seq(1,3)])
kable(ex2_cdc)
```

| ns | sigmab | Method | recovery A | mean rate A | recovery I | mean rate I | Time | 50% | 75% | 95% |
| --- | --- | --- | --- | --- | --- | --- | --- | --- | --- | --- |
| 40 | 0.1 | CDC-SIS | 0.37 | 0.82 | 0.31 | 0.808 | 0.813 | 224 | 470 | 799 |
| 80 | 0.1 | CDC-SIS | 0.895 | 0.974 | 0.762 | 0.941 | 5.214 | 54 | 167 | 503 |
| 100 | 0.1 | CDC-SIS | 0.958 | 0.989 | 0.865 | 0.966 | 9.917 | 30 | 94 | 337 |
| 40 | 0.9 | CDC-SIS | 0.022 | 0.415 | 0.018 | 0.401 | 0.815 | 608 | 790 | 948 |
| 80 | 0.9 | CDC-SIS | 0.272 | 0.756 | 0.208 | 0.736 | 5.223 | 402 | 594 | 873 |
| 100 | 0.9 | CDC-SIS | 0.525 | 0.864 | 0.385 | 0.82 | 9.881 | 316 | 530 | 866 |

### Example 3: table 3

To reproduce table 3, simply run the command

```
Rscript perform_analysis.R ex3 400 filename
```

Similarly to above, this gives the following table:

```
ex3_results <- readRDS("sim_examples/intermediate_results/ex3_fullrun.rds")
ex3_results[,-seq(1,3)] <- as.numeric(ex3_results[,-seq(1,3)])
kable(ex3_results)
```

| ns | sigmab | Method | recovery A | mean rate A | recovery I | mean rate I | Time | 50% | 75% | 95% |
| --- | --- | --- | --- | --- | --- | --- | --- | --- | --- | --- |
| 40 | 0.1 | GEES.ind | 0.382 | 0.821 | 0.238 | 0.781 | 0.036 | 276 | 587 | 916 |
| 40 | 0.1 | GEES.cs | 0.382 | 0.821 | 0.238 | 0.781 | 0.061 | 276 | 587 | 916 |
| 40 | 0.1 | GEES.ar1 | 0.42 | 0.836 | 0.198 | 0.77 | 0.061 | 306 | 607 | 909 |
| 40 | 0.1 | BCor-SIS | 0.092 | 0.614 | 0.043 | 0.583 | 0.753 | 558 | 805 | 958 |
| 40 | 0.1 | LS.intercept | 0.09 | 0.73 | 0.675 | 0.914 | 2.955 | 251 | 361 | 480 |
| 40 | 0.1 | LS.slope | 0.495 | 0.858 | 0.767 | 0.94 | 3.617 | 49 | 84 | 261 |
| 80 | 0.1 | GEES.ind | 0.865 | 0.966 | 0.588 | 0.897 | 0.072 | 90 | 218 | 728 |
| 80 | 0.1 | GEES.cs | 0.865 | 0.966 | 0.588 | 0.897 | 1.43 | 90 | 218 | 728 |
| 80 | 0.1 | GEES.ar1 | 0.905 | 0.976 | 0.507 | 0.877 | 1.059 | 112 | 276 | 835 |
| 80 | 0.1 | BCor-SIS | 0.555 | 0.884 | 0.31 | 0.818 | 4.103 | 294 | 561 | 873 |
| 80 | 0.1 | LS.intercept | 0.38 | 0.845 | 0.998 | 0.999 | 3.152 | 125 | 186 | 271 |
| 80 | 0.1 | LS.slope | 0.995 | 0.999 | 0.995 | 0.999 | 4.025 | 7 | 11 | 28 |
| 100 | 0.1 | GEES.ind | 0.935 | 0.984 | 0.762 | 0.941 | 0.106 | 48 | 152 | 585 |
| 100 | 0.1 | GEES.cs | 0.935 | 0.984 | 0.762 | 0.941 | 1.252 | 48 | 152 | 585 |
| 100 | 0.1 | GEES.ar1 | 0.955 | 0.989 | 0.66 | 0.915 | 1.279 | 66 | 186 | 659 |
| 100 | 0.1 | BCor-SIS | 0.72 | 0.929 | 0.422 | 0.853 | 6.33 | 234 | 494 | 845 |
| 100 | 0.1 | LS.intercept | 0.632 | 0.908 | 1 | 1 | 3.274 | 92 | 144 | 219 |
| 100 | 0.1 | LS.slope | 1 | 1 | 1 | 1 | 3.899 | 6 | 7 | 13 |
| 40 | 0.9 | GEES.ind | 0.155 | 0.682 | 0.098 | 0.654 | 0.03 | 405 | 688 | 930 |
| 40 | 0.9 | GEES.cs | 0.155 | 0.682 | 0.098 | 0.654 | 0.06 | 405 | 688 | 930 |
| 40 | 0.9 | GEES.ar1 | 0.155 | 0.676 | 0.08 | 0.634 | 0.067 | 432 | 690 | 952 |
| 40 | 0.9 | BCor-SIS | 0.018 | 0.431 | 0.013 | 0.41 | 0.736 | 650 | 831 | 964 |
| 40 | 0.9 | LS.intercept | 0.068 | 0.706 | 0.623 | 0.897 | 2.924 | 286 | 436 | 723 |
| 40 | 0.9 | LS.slope | 0.178 | 0.737 | 0.672 | 0.911 | 3.493 | 144 | 320 | 665 |
| 80 | 0.9 | GEES.ind | 0.708 | 0.926 | 0.422 | 0.853 | 0.069 | 184 | 441 | 850 |
| 80 | 0.9 | GEES.cs | 0.708 | 0.926 | 0.422 | 0.853 | 0.934 | 184 | 441 | 850 |
| 80 | 0.9 | GEES.ar1 | 0.735 | 0.931 | 0.348 | 0.832 | 0.979 | 230 | 517 | 884 |
| 80 | 0.9 | BCor-SIS | 0.29 | 0.771 | 0.162 | 0.728 | 3.076 | 438 | 693 | 951 |
| 80 | 0.9 | LS.intercept | 0.272 | 0.818 | 0.993 | 0.998 | 3.041 | 166 | 263 | 455 |
| 80 | 0.9 | LS.slope | 0.787 | 0.946 | 0.993 | 0.998 | 3.849 | 36 | 76 | 237 |
| 100 | 0.9 | GEES.ind | 0.843 | 0.961 | 0.578 | 0.892 | 0.106 | 120 | 340 | 859 |
| 100 | 0.9 | GEES.cs | 0.843 | 0.961 | 0.578 | 0.892 | 1.253 | 120 | 340 | 859 |
| 100 | 0.9 | GEES.ar1 | 0.863 | 0.966 | 0.46 | 0.863 | 1.213 | 168 | 429 | 878 |
| 100 | 0.9 | BCor-SIS | 0.47 | 0.857 | 0.28 | 0.799 | 5.161 | 382 | 622 | 905 |
| 100 | 0.9 | LS.intercept | 0.44 | 0.86 | 1 | 1 | 3.132 | 132 | 216 | 383 |
| 100 | 0.9 | LS.slope | 0.907 | 0.977 | 1 | 1 | 4.1 | 19 | 47 | 164 |

```
ex3_cdc <- readRDS("sim_examples/intermediate_results/ex3_cdcscreen.rds")
ex3_cdc[,-seq(1,3)] <- as.numeric(ex3_cdc[,-seq(1,3)])
kable(ex3_cdc)
```

| ns | sigmab | Method | recovery A | mean rate A | recovery I | mean rate I | Time | 50% | 75% | 95% |
| --- | --- | --- | --- | --- | --- | --- | --- | --- | --- | --- |
| 40 | 0.1 | CDC-SIS | 0.312 | 0.797 | 0.235 | 0.775 | 0.664 | 266 | 505 | 775 |
| 80 | 0.1 | CDC-SIS | 0.843 | 0.961 | 0.728 | 0.932 | 4.882 | 61 | 156 | 424 |
| 100 | 0.1 | CDC-SIS | 0.945 | 0.986 | 0.863 | 0.966 | 9.519 | 33 | 89 | 304 |
| 40 | 0.9 | CDC-SIS | 0.11 | 0.646 | 0.102 | 0.638 | 0.665 | 434 | 661 | 878 |
| 80 | 0.9 | CDC-SIS | 0.627 | 0.905 | 0.575 | 0.89 | 4.93 | 128 | 287 | 692 |
| 100 | 0.9 | CDC-SIS | 0.765 | 0.941 | 0.757 | 0.939 | 9.518 | 78 | 217 | 542 |

### Example 4: table 4

To reproduce table 4, run the command

```
Rscript perform_analysis.R ex4 400 filename omega
```

where omega is the between-variables correlation.

To reproduce Table 4, run the above command for omega = {0,0.5,0.9},
and run the following command with suitable file names:

```
files <- c("ex4_rho0_fullrun.rds", "ex4_rho05_fullrun.rds", "ex4_rho09_fullrun.rds")
methods <- c("GEES.ind", "GEES.ar1", "BCor-SIS", "LS.intercept", "LS.slope","CDC-SIS")

ex4_main_table <- methods
ex4_int_table <- methods
for (i in 1:length(files)){
  file <- files[i]
  ex4_results <- readRDS(paste0("sim_examples/intermediate_results/",file))
  ex4_results[,-seq(1,3)] <- as.numeric(ex4_results[,-seq(1,3)])
  ex4_results <- ex4_results[ex4_results[,1] == 80 & ex4_results[,2] == 0.1, c(3:4,6)]
  ex4_results <- ex4_results[-2,]
  ex4_main_table <- cbind(ex4_main_table, ex4_results[,2])
  ex4_int_table <- cbind(ex4_int_table, ex4_results[,3])
}

colnames(ex4_int_table) <- colnames(ex4_main_table) <- c("", "omega = 0", "omega = 0.5", "omega = 0.9")
```

To show the the first columns corresponding \(r\_M\) in Tabe 4, run:

```
kable(ex4_main_table)
```

|  | omega = 0 | omega = 0.5 | omega = 0.9 |
| --- | --- | --- | --- |
| GEES.ind | 0.833 | 0.627 | 0.56 |
| GEES.ar1 | 0.835 | 0.627 | 0.565 |
| BCor-SIS | 0.595 | 0.492 | 0.378 |
| LS.intercept | 0.4 | 0.165 | 0.135 |
| LS.slope | 0.998 | 0.948 | 0.912 |
| CDC-SIS | 0.853 | 0.485 | 0.39 |

and similarly for \(r\_I\):

```
kable(ex4_int_table)
```

|  | omega = 0 | omega = 0.5 | omega = 0.9 |
| --- | --- | --- | --- |
| GEES.ind | 0.593 | 0.507 | 0.372 |
| GEES.ar1 | 0.593 | 0.507 | 0.375 |
| BCor-SIS | 0.195 | 0.302 | 0.202 |
| LS.intercept | 0.998 | 0.89 | 0.8 |
| LS.slope | 0.998 | 0.948 | 0.912 |
| CDC-SIS | 0.818 | 0.485 | 0.372 |

### Real life data example

#### Stability screening: Figure 1

To reproduce Figure 1 in the manuscript, run the command (inside the
folder “data\_example”)

```
Rscript realdata_stabilityscreening.R 100
```

This will save an .eps figure called “histDistribution\_ne.eps”
corresponding to Figure 1. The number 100 is the number of bootstrap
samples, and this can be lowered to reduce running time.

#### Stability selection with SCAD: table 4 and 5

Please note: the tables 5 and 6 were run with

```
R version 4.3.3 (2024-02-29)
Platform: x86_64-apple-darwin20 (64-bit)
Running under: macOS Monterey 12.6
```

as opposed to the simulated examples and stability screening.

To reproduce table 5, run the command

```
Rscript realdata_stabilityselectionSCAD.R 1000
```

where the number 1000 is the the maximum number of iterations in the
SCAD estimation and this can be lowered for reduced running time. This
command will save a file called “screening\_overlap\_table.rds” which
reproduces table 5.

To see the results, run the command

```
overlap_matrix <- readRDS("data_example/intermediate_results/screening_overlap_table.rds")
kable(overlap_matrix)
```

|  | Likelihood screen | SIS | GEES.cs | GEES.ar1 | BCor-SIS | CDC-SIS |
| --- | --- | --- | --- | --- | --- | --- |
| Likelihood screen | NA | 12 | 29 | 6 | 12 | 18 |
| SIS | NA | NA | 8 | 0 | 27 | 42 |
| GEES.cs | NA | NA | NA | 8 | 5 | 10 |
| GEES.ar1 | NA | NA | NA | NA | 0 | 1 |
| BCor-SIS | NA | NA | NA | NA | NA | 35 |
| CDC-SIS | NA | NA | NA | NA | NA | NA |

The file “realdata\_stabilityselectionSCAD.R” will after many hours
(if max iterations is set to 1000) also save the final selected
variables for the 5 methods in the manuscript. This will be saved in the
file “stabilityselectionSCAD\_topvariables.rds” and can be used to
reproduce Table 6. To lower the running time, set max iterations to 100
and the results will not be very different form the results from 1000
iterations.

```
top_table <- readRDS("data_example/intermediate_results/stabilityselectionSCAD_topvariables_maxIter1000.rds")
```

LS.slope:

```
kable(top_table[["likeli"]][,-1])
```

|  | Freq | gene | type |
| --- | --- | --- | --- |
| 53 | 0.81 | BLNK | main |
| 27 | 0.80 | FCER1A | main |
| 55 | 0.80 | ITGA2B | main |
| 17 | 0.74 | ATG16L1 | main |
| 113 | 0.71 | GUSB | interaction |
| 63 | 0.68 | LILRA3 | main |
| 33 | 0.67 | HLA.DRB1 | main |

GEES.ar1:

```
kable(top_table[["ar1"]][,-1])
```

|  | Freq | gene | type |
| --- | --- | --- | --- |
| 89 | 0.85 | TRAF5 | main |
| 95 | 0.68 | sCTLA4 | main |
| 8 | 0.67 | CTLA4\_all | interaction |
| 20 | 0.67 | ICAM2 | main |
| 93 | 0.67 | ZEB1 | main |
| 112 | 0.64 | CD209 | main |
| 7 | 0.61 | CTLA4\_all | main |
| 29 | 0.60 | IL1RL2 | main |

BCor-SIS:

```
kable(top_table[["bcor"]][,-1])
```

|  | Freq | gene | type |
| --- | --- | --- | --- |
| 71 | 0.75 | LILRA3 | main |
| 106 | 0.74 | TNFRSF4 | main |
| 16 | 0.71 | FCER1A | main |
| 102 | 0.68 | TCF4 | main |
| 33 | 0.66 | HLA.DRB1 | main |
| 34 | 0.62 | HLA.DRB1 | interaction |
| 55 | 0.60 | KIR3DL2 | main |

Finally, to get the mean prediction error, run the code

```
library(lme4)
```

```
## Loading required package: Matrix
```

```
get_pred_error <- function(df,top_variables,i){
  set.seed(i)
  id.tot <- df$id
  ns <- length(unique(id.tot))
  df$t0 <- as.numeric(df$t)-1
  t1 <- as.numeric(sample(unique(id.tot),floor(0.8*ns),replace=F)) #training set
  t2 <- unique(id.tot)[!(unique(id.tot) %in% t1)] #test set
  df.t1 <- df[df$id %in% t1,]
  df.t2 <- df[df$id %in% t2,]
  form <- as.formula(paste0("y~",paste0(top_variables,collapse="+"),"+meal+t+(1|id)"))
  mod <- lmer(form, data = df.t1)
  yhat <- predict(mod, newdata = df.t2, re.form = NA)
  return(mean((yhat-df.t2$y)^2))
}


df <- readRDS("data_example/data/combineddata_deindentified.rds")

prediction_errors <- list()
for (method in c("likeli","ar1","bcor")){
  top_vars <- as.character(top_table[[method]]$res)
  pred_err <- sapply(1:100, function(k) get_pred_error(df,top_vars,i=101+k))
  prediction_errors[[method]] <- mean(pred_err)
}

pred_table <- unlist(prediction_errors)
names(pred_table) <- c("LS.slope", "GEES.ar1", "BCor-SIS")
kable(pred_table)
```

|  | x |
| --- | --- |
| LS.slope | 0.2856115 |
| GEES.ar1 | 0.3040321 |
| BCor-SIS | 0.2837690 |
