# Supplementary figures and images for: Conditional Variable Screening for Ultra‐High Dimensional Longitudinal Data With Time Interactions
Source: Biom J. 2024 Nov 23;66(8):e70005. doi: 10.1002/bimj.70005 (PMC11585226; doi:10.1002/bimj.70005)

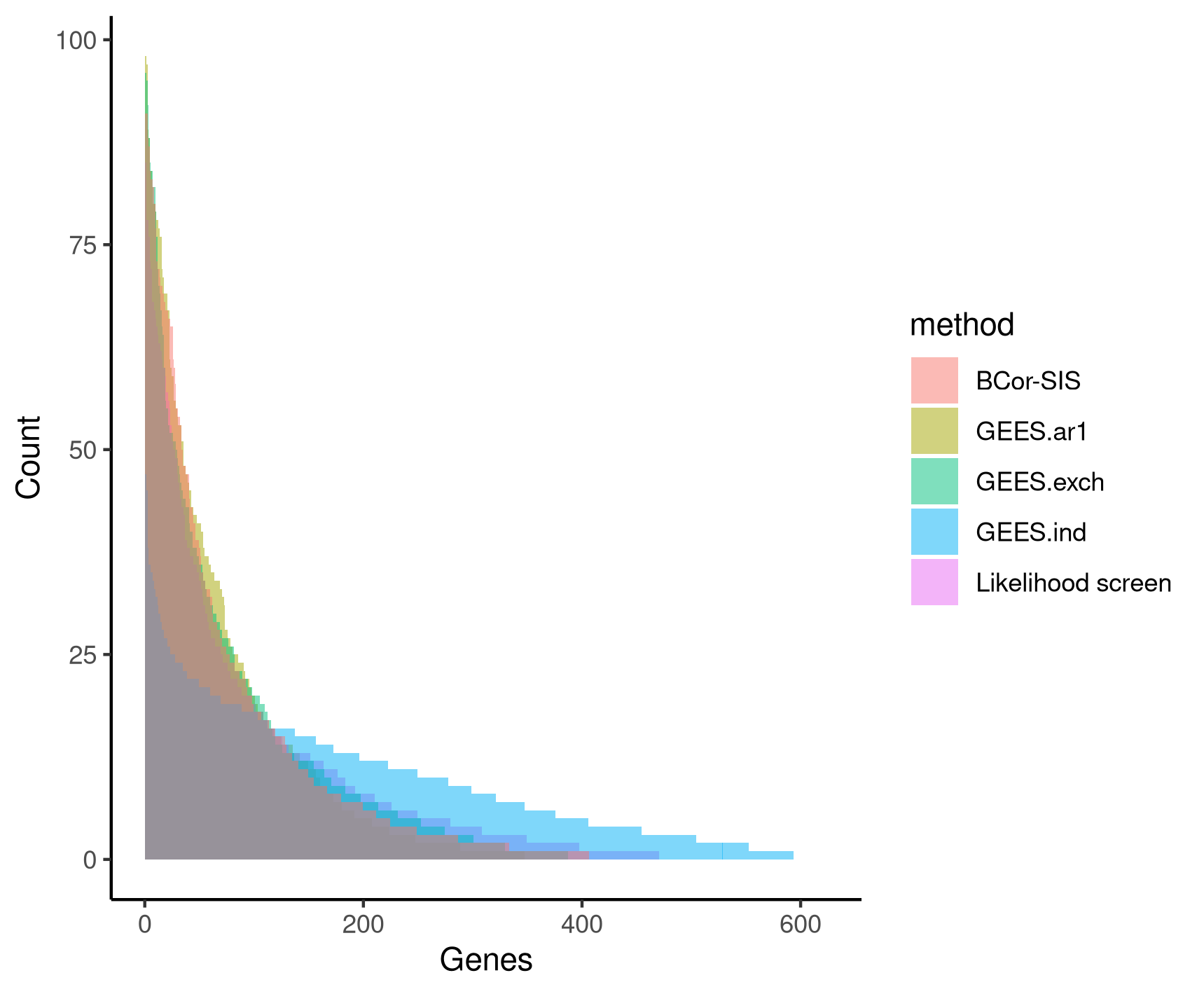

Supplement: Supplementary file 2 — Supporting Information [file BIMJ-66-e70005-s001.zip › code_supplement_bimj.202300154/data_example/figures/histDistribution_all_fullrun.tiff]
